# Lanolin

**Published:** 1886-10

**Authors:** 


### *LANOLIN.*

Lanolin is the name given to a new fatty substance obtained from the alkaline water-wastings of wool, and very highly recommended as a base for various medicated salves and ointments used in treating cutaneous eruptions and other external as well as internal disorders. It consists of varying proportions of cholesterin and fat acids, with which is incorporated a certain percentage of water, which it readily absorbs, thus forming a smooth, white unctuous mass. As some of our professional readers will be interested to know how it is prepared, we are able to present the following formula, as contained in a late issue of the *Western Druggist:*

"The wool-washings are first passed through a fine sieve to free them from mechanical impurities, and then through a convenient quantity of cut-straw or saw-dust ; the solution is then treated with magnesium sulphate, and the resulting magnesium soap, containing also the cholesterin, is collected, well washed with water, then drained and allowed to dry by exposure to air. It is then treated with sufficient diluted hydrochloric acid to decompose the soap ; a large excess of hydrochloric acid should be avoided, but sufficient added until a slight excess of acid is indicated, which is afterwards removed in the process. The resulting fatty scum, consisting of fatty acids and cholesterin, is drained and treated with petroleum benzine in a closed vessel, slightly warmed to about 85° F. to aid solution, and then filtered through flannel in a closed filter press. The petroleum benzine is then driven off by evaporation or distillation, and to remove any remaining traces of hydrochloric acid the residue is treated with from one-tenth to one-fourth per cent. of carbonate of magnesia, rubbed up with water, the mixture being then well washed with fresh portions of water until the water-washings are no longer milky from the presence of magnesium carbonate. It is again melted, filtered while hot through flannel, and, when cold, water is incorporated and the lanolin becomes white, hard, and smooth."

Prof. Oscar Liebreich, of Berlin, who was the first to introduce this new substance, and give it a name, does not appear to fully agree with the foregoing formula of Gawalovski. In a treatise recently read before the British Medical Association, he says: "In my opinion, the best method consists in separating the lanolin from its emulsion by centrifugal action. The emulsion must be previously sterilized by the action of heat and alkalies. Distillation can, unfortunately, not be used, as it causes a partial decomposition and an increase in the quantity of fatty acids. When lanolin was first introduced by me, I recommended that it should be mixed with fat; but I am pleased to be able to withdraw this opinion, as I have succeeded in removing from lanolin the sticky cholesterin fats." * * * "In order to give you an idea of the differences which exists between these two sorts of fat. I may mention that cholesterine fats are not decomposed by boiling with alkaline solutions—that is to say, they do not form soaps; while, on the other hand, glycerine fats are easily split into soap and glycerine when treated in the same way. * * * I have found yet another difference between these two sorts of fat. Lanolin absorbs water very rapidly. By simply stirring together a cholesterine fat and water, more than 100 per cent. of the latter can be incorporated with it, and a soft yellowish mass is formed, which is the lanolin, properly so-called." * * *

"I may mention also that the easy penetration of the skin by lanolin explains the good results obtained with it in seborrhœa sicca. It stands to reason that the permeability of the skin varies in different persons; it is evident also that an epidermis already saturated by its natural lanolin cannot be impregnated with more fat as easily as a dry epidermis. Dr. Pawlowsky has therefore recommended to free the skin from its natural fat by means of ether before using the lanolin. There is no doubt that gray ointments prepared with lanolin are preferable to the ordinary ointments. This is the opinion of the well-known authority in Aix-la-Chapelle, Dr. Brandis. * * *

"I must end with a few remarks on a point of importance concerning the use of lanolin, which shows very evidently the difference between it and ordinary fat. When lanolin is used on mucuous surfaces it never forms a scab; this has been, I believe, first noticed by B. Fraenkel, of Berlin. It may be explained in the following way: Lanolin has a great affinity for water, and this peculiarity explains why it adheres intimately to mucous membranes; fat and vaseline, on the contrary, remain separated from the mucous surface by their natural watery secretion.

"It would lead me too far if I were to consider in detail the distribution of lanolin in the body, but I have reason to believe that it is found not only in the keratinous tissues, but also in the blood, liver, and other organs; never in the pamisculus adiposus. Further researches will elucidate this question.

"It has been objected to me that lanolin, which was known to the ancients and used for centuries, would not have been abandoned if it had been really useful. To this I answer, that what they used was œsypus—that is to say, impure wool oil, which has many disadvantages and dangers. I cannot, therefore, lay too much importance on the fact that pure neutral lanolin must be used; not those substances which are sometimes falsely called lanolin, but which are in reality only impure acid wood oil, or products of distillation which contain still larger quantities of acids."

